# Supplementary material for: The causal role of circulating inflammatory markers in osteoporosis: a bidirectional Mendelian randomized study
Source: Front Immunol. 2024 Jul 18;15:1412298. doi: 10.3389/fimmu.2024.1412298 (PMC11291241; doi:10.3389/fimmu.2024.1412298)
Supplement: Supplementary file 1 [file DataSheet_1.zip › supplementary material/CIM-OSTEOPOROSIS /GCST90274826.csv_forest.pdf]

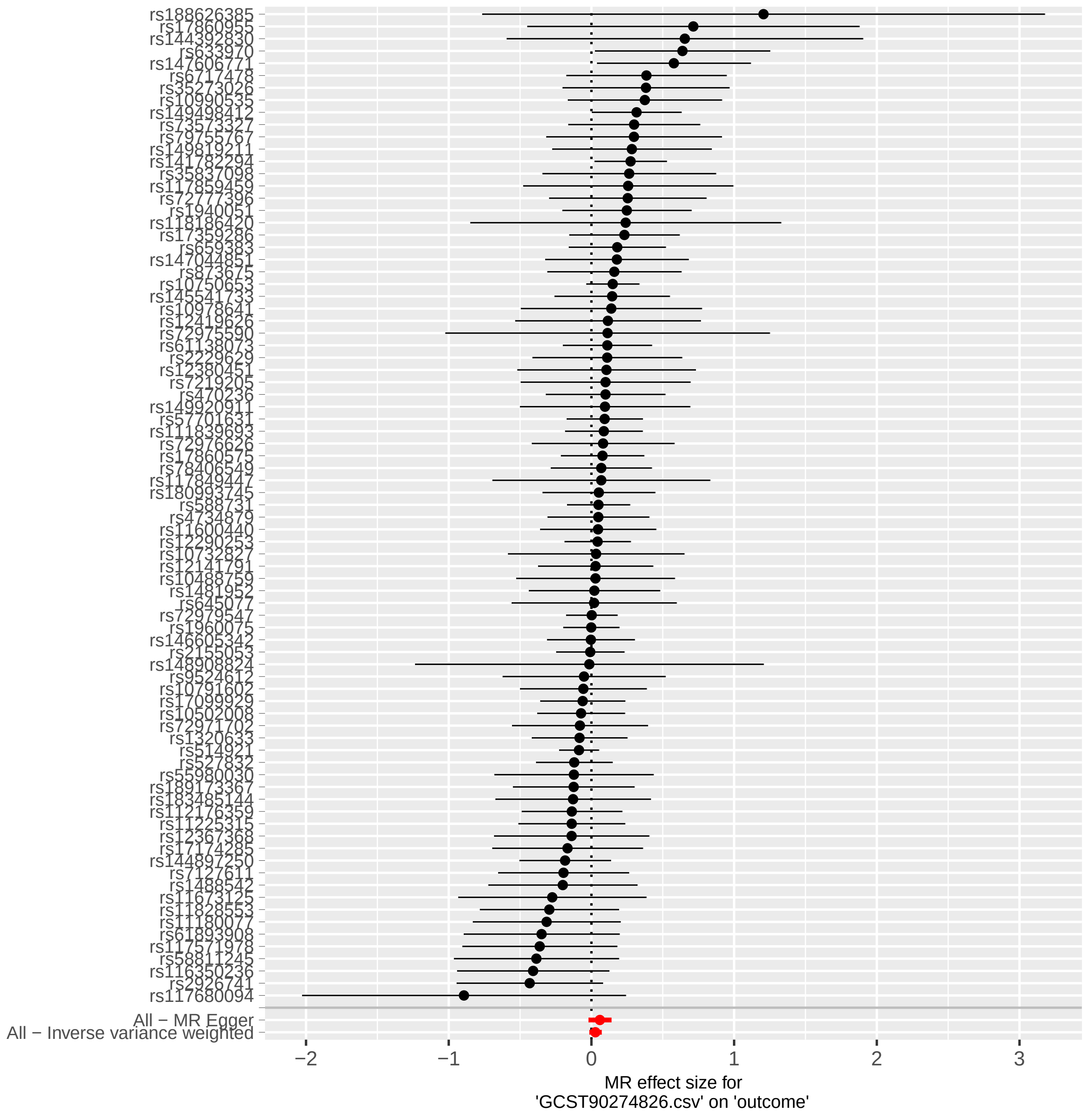
rs188626385
rs17860955
rs144392830
rs633970
rs147606771
rs6717478
rs35273026
rs10990535
rs149498412
rs73573327
rs79755767
rs149819211
rs141782294
rs35837098
rs117859459
rs72777396
rs1940051
rs118186420
rs17359286
rs659383
rs147044851
rs873675
rs10750653
rs145541733
rs10978641
rs12419626
rs72975590
rs61138073
rs2229629
rs12380451
rs7219205
rs470236
rs149920911
rs57701631
rs111839693
rs72976626
rs17860575
rs78406549
rs117849447
rs180993745
rs588731
rs4734879
rs11600440
rs12290253
rs10732827
rs12141791
rs10488759
rs1481952
rs645077
rs72979547
rs1960075
rs146605342
rs2155053
rs148908824
rs9524612
rs10791602
rs17099929
rs10502008
rs72971702
rs1320633
rs514921
rs527832
rs55980030
rs189173367
rs183485144
rs112176359
rs11225315
rs12367368
rs17174285
rs144897250
rs7127611
rs1488542
rs11673125
rs11828553
rs11180077
rs61893908
rs117571978
rs58811245
rs116350236
rs2926741
rs117680094
All − MR Egger
All − Inverse variance weighted
−2
−1
0
1
2
3
MR effect size for
'GCST90274826.csv' on 'outcome'
